# Congenital abnormalities associated with Zika virus infection–Dengue as potential co-factor? A systematic review

**DOI:** 10.1371/journal.pntd.0008984

**Published:** 2021-01-04

**Authors:** Stephanie Petzold, Nisreen Agbaria, Andreas Deckert, Peter Dambach, Volker Winkler, Jan Felix Drexler, Olaf Horstick, Thomas Jaenisch

**Affiliations:** 1 Heidelberg Institute of Global Health, Heidelberg University Hospital, Heidelberg, Germany; 2 Charité-Universitätsmedizin Berlin, corporate member of Freie Universität Berlin, Humboldt-Universität zu Berlin, Berlin Institute of Health, Institute of Virology, Berlin, Germany; 3 Martsinovsky Institute of Medical Parasitology, Tropical and Vector-Borne Diseases, Sechenov University, Moscow, Russia; 4 German Centre for Infection Research, associated partner site Charité, Berlin, Germany; 5 Section Clinical Tropical Medicine, Department for Infectious Diseases, Heidelberg University Hospital, Heidelberg, Germany; 6 Center for Global Health, Colorado School of Public Health, Aurora, Colorado, United States of America; Centre hospitalier de Cayenne, FRANCE

## Abstract

Zika virus (ZIKV) emerged in Brazil during 2013–2014 causing an epidemic of previously unknown congenital abnormalities. The frequency of severe congenital abnormalities after maternal ZIKV infection revealed an unexplained geographic variability, especially between the Northeast and the rest of Brazil. Several reasons for this variability have been discussed. Prior immunity against Dengue virus (DENV) affecting ZIKV seems to be the most likely explanation. Here we summarise the current evidence regarding this prominent co-factor to potentially explain the geographic variability.

This systematic review followed the PRISMA guidelines. The search was conducted up to May 15th, 2020, focussing on immunological interactions from Zika virus with previous Dengue virus infections as potential teratogenic effect for the foetus.

Eight out of 339 screened studies reported on the association between ZIKV, prior DENV infection and microcephaly, mostly focusing on antibody-dependent enhancement (ADE) as potential pathomechanism. Prior DENV infection was associated with enhancement for ZIKV infection and increased neurovirulence in one included in vitro study only. Interestingly, the seven in vivo studies exhibited a heterogeneous picture with three studies showing a protective effect of prior DENV infections and others no effect at all. According to several studies, socio-economic factors are associated with increased risk for microcephaly.

Very few studies addressed the question of unexplained variability of infection-related microcephaly. Many studies focussed on ADE as mechanism without measuring microcephaly as endpoint. Interestingly, three of the included studies reported a protective effect of prior DENV infection against microcephaly. This systematic review strengthens the hypothesis that immune priming after recent DENV infection is the crucial factor for determining protection or enhancement activity. It is of high importance that the currently ongoing prospective studies include a harmonised assessment of the potential candidate co-factors.

## Author summary

Despite the large volume of Zika virus (ZIKV)-related research, the difference in the frequency of ZIKV-related severe congenital abnormalities between Northeastern Brazil in the rest of Brazil and other locations has not been adequately explained. This systematic review summarizes the existing evidence for the role of prior Dengue virus (DENV) infections as co-factor to explain the variability in the frequency of ZIKV-related severe congenital abnormalities. Results differed significantly according to study type (e.g. in vitro versus in vivo studies), and no clear picture emerged with regard to prior DENV infections causing ZIKV-related severe congenital abnormalities. However, evidence from additional studies not included in this systematic review that focus on immunological interactions between DENV and ZIKV infections imply the potential that severe ZIKV disease is more frequent in those with prior DENV infections. Therefore, an assessment of background immunity against closely related viruses (e.g. the flavivirus family) needs to be included in future and ongoing longitudinal studies on ZIKV-related congenital abnormalities.

## Introduction

Zika virus (ZIKV) swept through most of Latin America and the Caribbean in 2015/16, but severe complications were mainly reported in the Northeast of Brazil and the urban centers bordering the Atlantic Coast of Brazil [1,2].

When officially reported data across Brazil were assessed, an unexplained variability of the frequency of microcephaly over geography [2], and over time [1] was demonstrated. In addition, it remains unclear why the country reporting the second highest number of ZIKV infections–Colombia–seemed to have a much lower rate of microcephaly cases, compared to Brazil [3].

Major cohort studies estimate the risk of congenital abnormalities in ZIKV infection of 4–10% [4–8]. However, some of the studies reported strikingly different estimates for other neurological abnormalities such as cerebral calcifications, hypoplasia of cerebral structures and ventricular enlargement [6,7]. In one cohort study carried out in Sao Paulo state (an area with lower transmission) no microcephaly was recorded. The disease outcomes in this cohort study were sub-ependymal cysts, auditory disorders or chorioretinitis, which supports the claim of a broader definition for the congenital Zika syndrome (CZS) [9].

Officially reported data in Brazil showed considerable heterogeneity between the first wave of ZIKV infections in 2015 with higher numbers of congenital complications (notable microcephaly) compared to the second wave in 2016 [10,11]. The geographical and temporal variability of severe complications has puzzled many researchers, prompting the search for potential co-factors [12]. The role of co-factors was also highlighted in 2016 when an WHO expert panel assessed the causal association between ZIKV infection and severe neurological complications [13]. Since then, a number of co-factors have been suggested in the literature–among them socio-economic status, prior Dengue virus (DENV) infections, (environmental) toxins used for vector control, vaccine administration during pregnancy, and other teratogenic infectious agents (TORCH) [14–17].

Some researchers have argued that this variability may at least partly result from inconsistent terminology, difficulties in the accurate measurement of microcephaly and unreliability of secondary data sources [3,10,18].

Even though DENV is endemic all over Brazil, the transmission intensity, the timing of local outbreaks, and the mix of serotypes differs geographically. Antibody dependant enhancement (ADE) has been suggested to explain a higher risk of severe Dengue in secondary infections caused by a heterologous serotype compared to primary infections [19]. Because of the close phylogenetic relationship between DENV and ZIKV, ADE is hypothesized to play a role as well for congenital abnormalities after maternal ZIKV infection during pregnancy. Many researchers suspect that the unbalanced distribution of severe congenital abnormalities after maternal ZIKV infection is associated with prior DENV infections [20,21], because of the immunological interactions caused by sequential infections by the four DENV serotypes and by ZIKV [22,23].

## Methods

We conducted this systematic literature review following a pre-defined research protocol registered in the Open Science Framework database and in accordance with the Preferred Reporting Items for Systematic Reviews and Meta-Analyses (PRISMA) guidelines [24]. We aimed to include studies of ZIKV positive pregnant women that also included DENV infection as effect modifier for microcephaly or congenital abnormalities as endpoint of interest. Inclusion criteria were: 1) studies which had Zika and Dengue as condition or explained potential interactions of Zika, Dengue, and microcephaly, 2) no limitations in the search mask, 3) human or non-human primate studies, 4) studies in English, Portuguese or Spanish. There was no restriction on the year of publication. Exclusion criteria were 1) clinical trials, 2) opinion papers/reports.

We searched Cochrane, Google scholar, LILACS and PubMed online databases until the 15^th^ of May 2020. A broad search was carried out with the key terms “Zika” AND “Dengue” AND either “microcephaly” or “congenital abnormalities”. We used the COVIDENCE web-based software platform as a literature management tool. Titles and abstracts of the identified references were screened by two authors against the inclusion/exclusion criteria. Any discrepancies between the screening determinations were resolved between the reviewers by consensus.

Study quality assessment (S2 Quality assessment) was applied, using a modified tool of the Joanna Briggs approach [25]. We used a combined version of the study specific tools, to harmonise our different study types and make them comparable. The assessment has been used to weight the results of different studies in its context. Middle and high-quality studies were presented as such. We planned for exclusion of low-quality studies.

## Results

**Fig 1:**
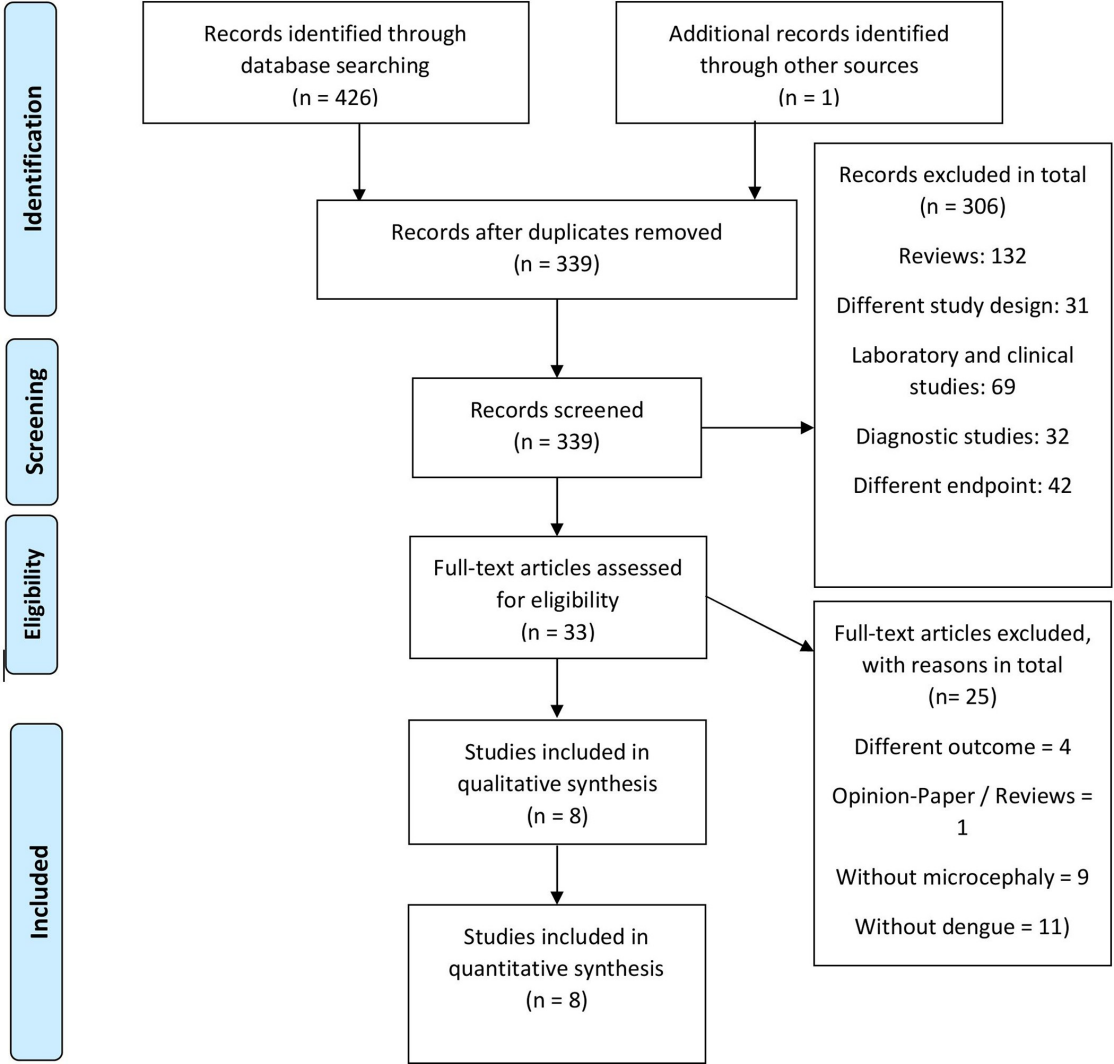
Prisma 2009 Flow Diagram.

We identified 339 non-duplicate articles (Fig 1). When applying inclusion and exclusion criteria, 33 articles were fully reviewed, of which 8 met all criteria for inclusion (Table 1). No study was excluded because of quality assessment since all scored middle and high. The included articles highlighted the association between ZIKV and prior DENV infection, mostly referring to the mechanism of antibody-dependent-enhancement (ADE).

**Table 1:** Listed studies through Systematic Review.

First Author	Sample size	Study design	Diagnostic test	Time frame	Location	Results	
						pro antibody-depended enhancement	contra antibody-depended enhancement
Moreira-Soto et al., 2017	N cases = 28 N controls = 122	In vivo, Case-Control	PRNTs and ELISA	October 2015 –December 2016	Salvador de Bahia, Brazil		**Reduced Dengue virus (DENV) antibody status in microcephaly cases** compared to controls
Moreira-Soto et al., 2018	N cases = 32 N controls = 160	In vivo, Case-control	PRNTs and ELISA	May 2015-October 2016	Salvador de Bahia, Brazil		DENV antibody status **did not differ between microcephaly cases and controls**
Pedroso et al., 2019	N cases = 29 N controls = 108	In vivo, Case-control	PRNTs and ELISA	May 2015-December 2016	Salvador de Bahia, Brazil		**Strong cross-protection** by DENV- immunity for congenital zika syndrome
Halai et al., 2017	N = 121	In vivo, Prospective cohort	RT-PCR	September 2015- May 2016	Rio de Janeiro, Brazil		**No effect** of prior DENV antibody status on Zika virus (ZIKV) clinical severity or abnormal birth outcome
Castanha et al., 2019	N cases = 89 N controls = 173	In vivo, Case-Control	PRNTs, RT-PCR, ELISA	January–December 2016	Recife, Brazil		**No difference** between cases and controls regarding ZIKV and DENV infection
Campos et al., 2018		Ecological study (Secondary data: Health Informatics Department of the Brazilian Ministry of Health)		2014–2016	Brazil		**No overlap** in distribution between DENV, ZIKV, and microcephaly
Carvalho et al., 2020		Ecological study (Secondary data: Health Informatics Department of the Brazilian Ministry of Health)		DENV 2001–2014 Microcephaly 2015/2016	Brazil	**enhancement activity** (7 years post DENV infection)	**protection** (up to 6 years prior DENV infection)
Rathore et al., 2019		In vitro, Mouse model	PCR and ELISA	ZIKV H/PF/2013 strain	French Polynesia	Prior immunity to dengue **can enhance** ZIKV infection	

### Immunological interactions with prior DENV infection

Of the eight included studies that focused on prior DENV infections, seven were in vivo and one in vitro study. Interestingly, within the in vivo studies, three papers described a protective effect of prior DENV infections against symptomatic ZIVK infection. The remaining in vivo papers did not detect an association between prior DENV antibody status and ZIKV infection, severity or congenital abnormalities. The one in vitro study described an enhancement effect of prior DENV infections and ZIKV infection with microcephaly.

Three case-control studies carried out in Salvador de Bahia, Brazil, were included. In one of the case-control studies, Moreira-Soto et al. found that a prior DENV infection was not associated with higher ZIKV-specific plaque reduction neutralization test (PRNT) titers in cases–on the contrary, the presence of anti-ZIKV antibodies was associated with an absence of microcephaly [26]. The authors conducted a second nested case control study, also in Bahia, where the presence of DENV antibodies was again associated with the absence of abnormalities, although not statistically significant [17]. A third nested case control study conducted from Pedroso et al. [14] was included. The results highlighted that DENV antibody titers did not differ between cases and controls. The results rather indicated a strong cross-protection, especially for serotypes DENV2 and DENV4. Besides, the analyses of historical DENV genomic data indicated no evidence for a unique DENV serotype in the Northeast, which would be an explanation for the higher rates of infection-related microcephaly in this region. It needs to be noted that the subjects studied overlapped to a large degree in all three studies.

Halai et al. [27] studied 131 Zika-PCR positive pregnant women presenting for medical care in Rio de Janeiro with new onset rash. A standardized ZIKV infection clinical severity score was developed in order to distinguish between levels of disease severity. Most participants enrolled were in the second trimester of pregnancy. Prior DENV exposure was determined by a single serological assay and was neither associated with abnormal birth outcomes nor with a higher maternal ZIKV viral load or clinical severity.

Castanha et al. [28] explored in a case-control study the immunological profile among pregnant women in Recife, Brazil. One case with microcephaly was matched with two controls without microcephaly. As expected, there was a slightly higher ZIKV neutralizing antibody rate among mothers of cases than that of mothers of the controls. However, DENV exposure was detected in 85.8% of all mothers, similar in cases (p = 0.150) and controls (p = 0.414).

Campos et al. [15] analysed reported cases for Chikungunya, Dengue and Zika in an ecological study. They did not describe a correlation between the numbers of Zika reported cases per inhabitants and the distribution of infection-related microcephaly, nor an overlap between the distribution of microcephaly and Dengue. According to Campos et al., only Chikungunya prevalence showed a predominance in Northeast region of Brazil, which overlapped with the higher burden of microcephaly in general.

A second ecological study was included. Carvalho et al. [29] studied the interaction between Dengue fever epidemics and ZIKV-related microcephaly. The authors detected low Dengue fever incidence rates in areas with high microcephaly rates. Adjusting for time lag of previous Dengue infections, a protective effect of up to six years and an increasing risk of above seven years could be hypothesized. Of particular note, the authors suggested, that the prior Dengue fever epidemic effect was not strong enough to fully explain the variation in microcephaly incidence. One main limitation of this study is, that the authors could not control for Zika incidence. However, the higher rate of microcephaly triggered by ZIKV was recognized [1,7,16].

Contrary to the findings of the in vivo studies—which detected no or rather a protective effect, the in vitro study detected and described enhancement activity between ZIKV and DENV. Rathore et al. [30] used a mouse model with pregnant mice that were either naïve (to both ZIKV and DENV) or immune to DENV2. Mice were infected with ZIKV in the third semester of pregnancy (comparable to the assumed scenario in humans). Maternal antibodies enhanced ZIKV infection of the fetuses and lead to an exacerbated phenotype consistent with microcephaly, also characterized by a reduced cortical thickness and substantial loss of certain cortical layers that are crucial for brain development. The limitation of this study was the focus on one DENV serotype only (DENV2).

## Discussion

This systematic review yielded eight studies that addressed Dengue as potential co-factor for ZIKV infection related congenital abnormalities. Most studies focused on either antibody-dependent enhancement (ADE) as potential pathomechanism, or on cross-protective mechanisms induced by prior DENV infections. The findings differ significantly between in vivo and in vitro data. Enhancement after prior DENV infection was only observed in in vitro studies. Interestingly, in vivo studies showed a mixed picture with some in vivo studies reporting a protective effect of prior DENV infections while others report no effect at all.

In general, the large body of literature about in vitro ADE does not include microcephaly or congenital abnormalities as outcomes, but operates under the assumption that enhancement is associated with disease severity. For this reason, this literature is not formally included in this review—however it does constitute a potential link between epidemiological and in vitro data—firstly, because of its role as assumed underlying pathomechanism for more severe course of disease via higher viral load; secondly, because of the Fc *γ*-receptor mediated activity which is essential for the transplacental transfer of IgG.

Examples of in vitro studies not included in this systematic review because of our inclusion and exclusion criteria include Londono-Renteria et al. [31], Dejinirattisai et al. [32] and Castanha et al. [20]. These authors described enhancement activity between ZIKV and DENV in vitro, but do not report a direct link with microcephaly or congenital abnormality. From their results, it seems likely that heterogenous anti-DENV antibody-titers are associated with enhancement of the subsequent immune response against ZIKV, which in turn could be associated with more severe disease.

The enhancement between subsequent infections has long been described between heterogenous DENV serotypes. Katzelnick et al. [33] studied a cohort in Nicaragua, where children with moderately high pre-existing DENV antibody titers (1:21 to 1:80) exhibited a two-fold risk to develop Dengue shock syndrome compared to children with low titers (cumulative hazard: 11.4% to 6.6%). Interestingly, the risk decreased again (to the level of DENV naïve children) when the DENV antibody titers were very high (>1:1280). Thus, the relationship is non-linear and highlights that the timing of prior infection may be a crucial factor for determining protection vs. enhancement. In the case of DENV infections, it has been reported that the length of the time interval between sequential infections (with heterologous serotypes) plays a crucial role for enhancement [34–36].

Castanha et al. also described differences in enhancement mechanisms depending on the interval between either primary or secondary DENV infection and the subsequent ZIKV infection. Enhancement was not observed during the acute phase of the ZIKV infection after primary DENV infection, but at later time points. In addition, the enhancement effect for subsequent ZIKV infection was observed to be differential according to prior DENV serotype [31]. In line with these findings are the recently published results from Carvalho et al. [29] which described a protective effect from prior Dengue infection for up to six years, and an increase of risk starting 7 years post infection. Moreover, Pedroso et al. reported the strong cross-protection for DENV 2 and DENV4 serotypes, which were the most recent serotypes in the area around Bahia in Northeastern Brazil. These findings are supported by the studies from Gordon et al. and Rodriguez-Barraquer et al., in which pre-existing DENV immunity significantly reduced the risk for symptomatic Zika virus infection [21,37]. However, Santiago et al. article did not observe in vivo enhancement of ZIKV by anti-DENV antibodies, but did observe enhancement between sequential DENV infections [38].

An underlying assumption in the studies about ADE between DENV and ZIKV is that an increase in ZIKV loads is associated with severity of disease, and subsequently with the risk of severe abnormalities [12]. Although formally not included in our review as there was no association with microcephaly or congenital abnormalities described, there were three in vitro papers which studied the impact of cross-reactive DENV antibodies on ZIKV infection investigating placenta tissue. They provided evidence for enhancement as pathomechanism relevant for Zika pathogenesis. Their data showed that antibodies against DENV increased the breakdown of the placenta structure during ZIKV infection, leading to higher ZIKV infection rate, and promoting replication of the virus. The increased placental infection rate is hypothesized to correlate with an increase of fetal malformations [39–41]. On the other hand, contrary to these in vitro studies, Halai et al., did not find evidence in their prospective cohort for an association between disease severity and viral load, nor for disease severity and abnormal outcomes [27].

Interestingly, the two ecological studies, selected through this review, agreed that prior DENV infection should not have been the only cause for the variability in microcephaly incidence. However, ecological studies are not the best study design for epidemiological analysis of causal associations. This highlights the need for prospective studies to acquire an in-depth understanding of the role of previous DENV exposure on ZIKV infection.

Some researchers refuted the need for co-factors altogether to explain the variability of the burden of microcephaly. They suggested for example that the different frequency of severe complications between regions of Brazil can be explained by a higher transmission intensity translating to an increased case load of ZIKV infected cases in the Northeast of Brazil [42]. If true, this would translate into higher post-epidemic ZIKV seroprevalence rates in these regions, which could be empirically validated. Another potential explanation that refutes the role of co-factors is that the variability of severe abnormalities observed over geography and time is based on biased reporting and the variability of definitions, thus actually an artefact [18]. In this context, researchers highlighted that the diagnostic criteria for microcephaly were relatively unspecific [43]. Indeed, the Intergrowth and Fenton growth standard curves were often used interchangeably–however, Tuzun et al. compared both physical examination methods and found that the proportion of babies classified as ‘small for gestational age’ was significantly higher with the Intergrowth charts compared with the Fenton standard [44].

Although not formally included as it is likely a composite of more than one risk factor, socio-economic status (SES) or poverty was implicated with severe abnormalities in several studies. The Northeast of Brazil is one of the poorest regions of Brazil and had the highest microcephaly rates. Poverty increases the risk of malnutrition, which goes with poor general health. Possible mechanisms for SES to mediate severe abnormalities include higher prior exposure rates to the other co-factors (e.g. to mosquito-transmitted diseases like Dengue, Chikungunya) or reduced access to abortion services. The accumulation of garbage and rainwater storage could have led to an increase of *Aedes aegypti* populations and thus higher transmission rates in poor neighbourhoods. Studies reported a high frequency of ZIKV positivity among DENV-immune mothers which might reflect the high risk of this population to the exposure to *Aedes aegypti* and arthropod-borne virus infection [29,45].

## Conclusions

This systematic review focused on prior DENV infection as a co-factor to explain the variability of severe abnormalities in newborns after maternal ZIKV infection during pregnancy.

Results differed significantly according to study type (e.g. in vitro versus in vivo studies). Prior DENV infection was associated with enhancement for ZIKV infection and increased neurovirulence in one included in vitro study only—however, the seven in vivo studies exhibited a heterogenous picture with three in vivo studies showing a protective effect of prior DENV infections and others no effect at all. Evidence from additional studies that focus on immunological interactions between DENV and ZIKV infections imply the potential of enhancement being associated with more severe disease, but did not link enhancement to congenital abnormalities in ZIKV infections. If present, this association is time-dependent with a window of cross-protection and subsequently increased risk of enhancement, depending on the time interval and DENV antibody levels. This systematic review also highlights, that the variability of microcephaly and other neurological abnormalities after ZIKV infection is not well understood and needs to be further addressed. In the absence of other explanations for the variability of severe abnormalities across geography and over time, immunological interactions between DENV and ZIKV are still worth to be investigated in future studies [46]. An assessment of background immunity against related flaviviruses (including vaccination history against Yellow Fever and Dengue) needs to be included in future longitudinal studies, which need to carefully discuss the specificity of anti-DENV antibodies. In addition, it is important to better understand the biological pathomechanism of congenital abnormalities caused by maternal ZIKV infection in order to corroborate the roles of potential co-factors and conduct more targeted prospective studies.

## Supporting information

S1 PRISMA Checklist (DOC)

S1 Quality Assessment Table (DOCX)
