# Supplementary material for: Congenital abnormalities associated with Zika virus infection–Dengue as potential co-factor? A systematic review
Source: PLoS Negl Trop Dis. 2021 Jan 4;15(1):e0008984. doi: 10.1371/journal.pntd.0008984 (PMC7808571; doi:10.1371/journal.pntd.0008984)
Supplement: S1 Quality Assessment Table — (DOCX) [file pntd.0008984.s002.docx]

**S1 Quality Assessment**

| First author, Year | Study quality | | | | Adequate measurement of exposure | | |
| --- | --- | --- | --- | --- | --- | --- | --- |
|  | Study design | N Cases | Comparator Group, Recruitment, Outcome definition? | Methods presented in a reproducible way? | ZIVK exposure?  *(PCR / PRNT)* | Dengue exposure?  *(PCR / PRNT/ ELISA)* | Microcephaly measured?  *(Intergrowth Chart WHO or Fenton growth chart as standard)* |
| Moreira-Soto, 2017 | Case-Control | 28 | Yes | Yes | Yes | Yes | Yes |
| Campos, 2018 | Ecological study |  | Yes | Yes | Yes | Not clearly described | Yes |
| Rathore, 2019 | In vitro |  | Yes | Yes | Yes | Yes | Yes (for mice) |
| Moreira-Soto, 2018 | Case-Control | 32 | Yes | Yes | Yes | Yes | Yes |
| Carvalho, 2019 | Ecological study |  | Yes | Yes | No | Not clearly described | Yes |
| Castanha, 2019 | Case-Control | 89 | Yes | Yes | Yes | Yes | Yes |
| Pedroso, 2019 | Case-Control | 29 | Yes | Yes | Yes | Yes | Yes |
| Halai, 2017 | Prospective Cohort | 121 | Yes | Yes | Yes | Yes | Not clearly described |
